# FASCIA Method in the Assessment of Lymphocyte Mitogen Responses in the Laboratory Diagnostics of Primary Immunodeficiencies

**DOI:** 10.1007/s10875-022-01417-z

**Published:** 2022-12-13

**Authors:** Pauliina Lusila, Anne Toivonen, Hanna Jarva, Kim Vettenranta, Sari Lehtimäki, Eliisa Kekäläinen

**Affiliations:** 1 Translational Immunology Research Program, University of Helsinki, Helsinki, Finland; 2 Pediatric Research Center, Children’s Hospital, Helsinki University Hospital, University of Helsinki, Helsinki, Finland; 3 HUS Diagnostic Center, HUSLAB Clinical Microbiology and Immunology, Helsinki University Hospital, Helsinki, Finland; 4 HUS Diagnostic Center, HUSLAB Special Hematology, Helsinki University Hospital, Helsinki, Finland

**Keywords:** Combined immunodeficiency, severe combined immunodeficiency, diagnostic method, inborn errors of immunity, mitogen stimulation method, lymphocyte responses to mitogens, in vitro functional testing

## Abstract

Lymphocyte responses to mitogens constitute a key part of the diagnostics of combined immunodeficiency (CID). Currently, mostly radioactive thymidine incorporation and carboxyfluorescein diacetate succinimidyl ester (CFSE) dilution methods are used. Flow-cytometric assay for specific cell-mediated immune-response in activated whole blood (FASCIA) has been put forth as an easy-to-perform option for the measurement of lymphocyte responses with the advantage of recognizing different lymphocyte subtypes and avoiding the use of radioactive reagents. Our aim was to analyze retrospectively the usefulness of FASCIA in the diagnostics of CID. We included all lymphocyte stimulation tests done with FASCIA in HUSLAB (Helsinki, Finland) between February 2015 and September 2018 in our analysis. The cohort was divided into two groups according to the patients’ final diagnoses: CID (*n* = 30) or non-CID (*n* = 159). We evaluated the stimulation responses with a combined FASCIA score (the average of all mitogen responses). The FASCIA score was significantly lower among the CID group compared to the other patients (*p* = 0.002), and in the ROC analysis, the AUC was 0.75 (*p* < 0.001) for the FASCIA score. When the three mitogens were analyzed separately, phytohemagglutinin (PHA) was best in separating patients with CID from non-CID (in the ROC analysis AUC 0.71, *p* = 0.001). Immunosuppressive medication affected the FASCIA result significantly and needs to be considered when evaluating the results. In conclusion, FASCIA can reliably detect the CID patients in the absence of immunosuppressive medication. It emerges as a method with many benefits compared to tests requiring radioactive reagents or the complicated CFSE staining.

**Supplementary Information:**

The online version contains supplementary material available at 10.1007/s10875-022-01417-z.

## Introduction

Inborn errors of immunity are a large and heterogeneous group of diseases. Currently, more than 400 different entities are recognized as primary immunodeficiencies, and the number is growing [1]. Combined immunodeficiencies (CID) including severe combined immunodeficiency (SCID) are the most severe primary immunodeficiencies where impaired T cell function is a key feature. Accordingly, a reduced proliferation of lymphocytes in response to mitogens or T cell receptor stimulation is one of the diagnostic criteria of CID [2, 3]. Functional testing of lymphocytes is especially important in cases where the lymphocyte numbers are only slightly reduced or in the absence of a genetic diagnosis. However, measuring lymphocyte function remains a challenge. A test needs to discriminate CID patients from patients with milder immunodeficiencies or healthy individuals, and, in addition, robustness, ease-of-use, and cost are important factors when considering the practical application for clinical diagnostics.

Radioactive thymidine incorporation has been the golden standard in measuring lymphocyte function in clinical practice since its development in the 1950s, but its performance as a screening tool for CID diagnosis has not been studied widely. In a retrospective study analyzing years 1996–2003, thymidine incorporation showed an adequate discrimination for a broadly defined CID especially with Concavalin A (ConA) as the stimulus [4]. However, it is not sensitive enough in predicting opportunistic infections [4]. The thymidine incorporation method is dependent on radioactive reagents necessitating special requirements for the laboratory and poses an occupational safety issue. Furthermore, in comparison with the more modern flow cytometry-based methods, thymidine incorporation does not enable analyszng the T cell subpopulations.

Lymphocyte proliferation can also be monitored using a dye dilution method where cells are covalently loaded with carboxyfluorescein diacetate succinimidyl ester (CFSE) or similar commercial preparations prior to in vitro stimulation, and the following cell divisions can be measured with reduction in the dye intensity with flow cytometry [5]. A major disadvantage is that CFSE is toxic to the cells and negatively affects their proliferative capacity even in low concentrations. Thus, it may cause false positive results if the cells were to divide poorly due to the CFSE, instead of their poor intrinsic capacity to divide [6, 7]. The analysis of CFSE dilution is more complicated than with thymidine incorporation, but it enables a more detailed analysis of different lymphocyte populations through flow cytometry. However, the diagnostic performance of the CFSE method has not been formally analyzed in diagnosing CID.

A Swedish research group introduced the flow-cytometric assay for specific cell-mediated immune-response in activated whole blood (FASCIA) for the clinical diagnostics of SCID in 2014 [8]. In this study, they showed that FASCIA had a good agreement with the thymidine incorporation test in four SCID patients and 100 healthy controls. The authors concluded that FASCIA is reliable and easy to perform in the evaluation of lymphocyte function in patients.

There are a few major differences between FASCIA and the previous methods in measuring lymphocyte proliferation. In FASCIA, the activation of lymphocytes is measured as an increase in the cell size and no toxic chemicals like radioactively labeled thymidine or covalent cell labels are used. As with thymidine incorporation, CFSE method often requires mononuclear cell isolation prior to the staining and therefore a relatively large blood volume. Full-blood stimulation methods require less sample volume and are therefore much more suitable for pediatric patients. Moreover, as with CFSE assays, with FASCIA, it is possible to separate between the activation of different lymphocyte subtypes.

Our aim was to analyze retrospectively the diagnostic performance of FASCIA in the diagnostics of CID in a single-center retrospective patient cohort. We also evaluated if the patients’ immunosuppressive medication affected the FASCIA results. Our patient cohort consisted of both pediatric and adult patients evaluated for suspected immunodeficiency.

## Materials and Methods

### Study Design and Patient Cohorts

**Fig. 1:**
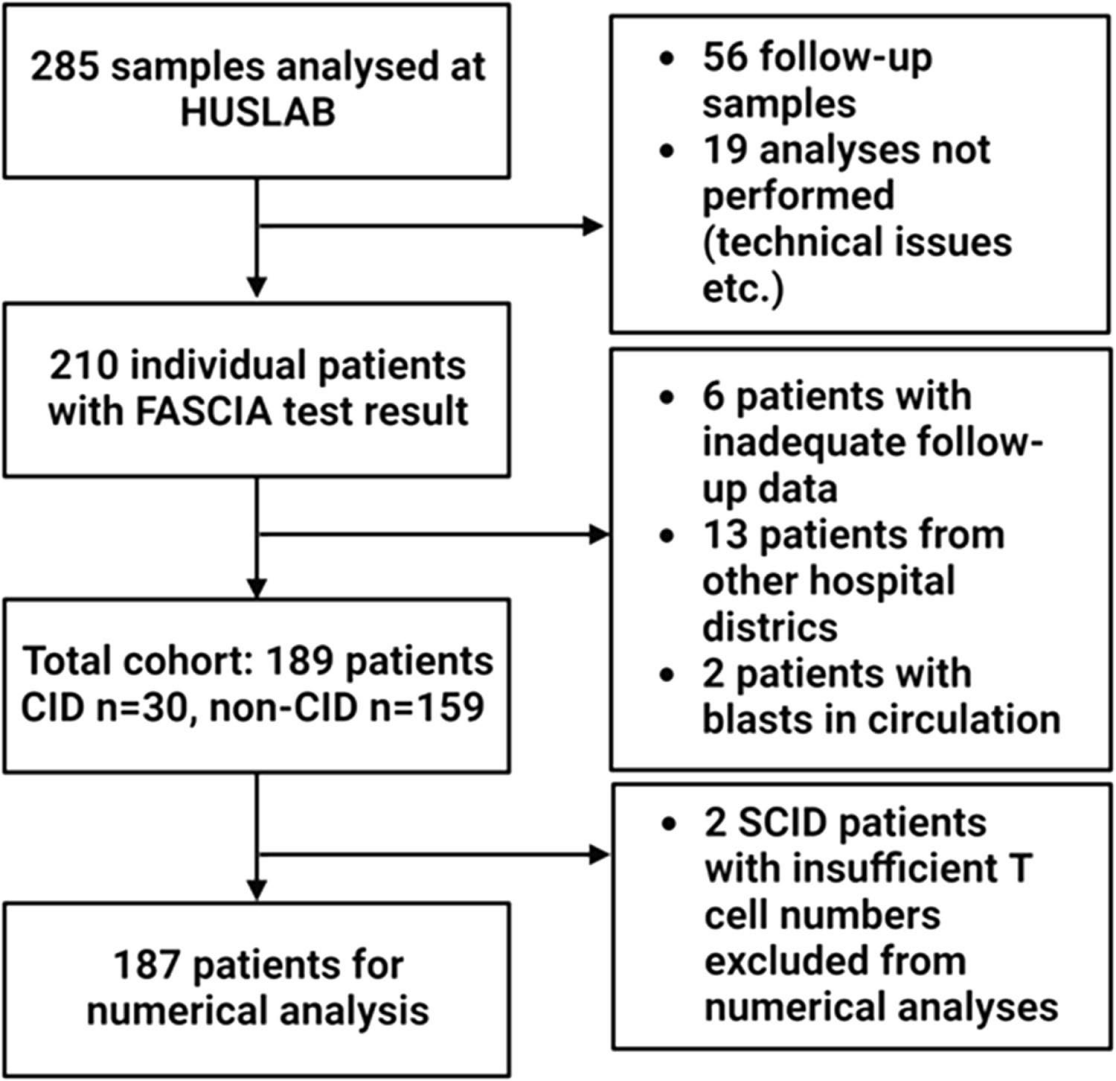
Flowchart of the sample and patient selection

This study was a retrospective, register-based analysis. The study was approved by the Research Administration of Helsinki University Hospital (HUS/157/2020). Data on all the lymphocyte stimulation tests (*n* = 285) performed in HUSLAB, HUS Diagnostic Center, from February 2015 to September 2018 were obtained from the laboratory information system (Fig. 1). Nineteen tests were excluded because of technical problems (e.g., the control sample stimulated in the same batch did not pass quality control, or the sample was stored for over 27 h before analysis). Some patients were tested more than once. In these cases, the first test result was included, and the follow-up samples (*n* = 56) were excluded. We also excluded analyses on patients treated outside the Hospital District of Helsinki and Uusimaa (*n* = 13) because no sufficient clinical information was available as well as those with inadequate follow-up data (*n* = 6). Two patients had an active hematological malignancy at the time of testing, and these analyses were also excluded. These patients form the study cohort (*n* = 189). Of the 7 SCID patients in the study cohort, one had no T or B lymphocytes, and two had so few lymphocytes that the test result could not be given numerically but could be clearly stated as abnormal. These patients could not be included in the final numerical analyses. A total of 186 analyses from 186 individual patients were included in the final analysis. The flowchart of the sample and patient selection is shown in Fig. 1.

The stimulation results from 169 healthy adult blood donors were used as controls. When performing a FASCIA stimulation, our laboratory always includes one blood sample from a healthy anonymous blood donor (Finnish Red Cross Blood Service) as a technical control. The control stimulations used were analyzed during the same period as the patient samples.

During the study period, there was a phase when the ConA mitogen could not be obtained, and the test was done without ConA (14 patients, 6 controls). Because of this, the total FASCIA result was not available for all the 189 patients.

### Data Collection and Patient Cohort Description

We collected the patient clinical data manually from the Helsinki University Hospital’s records. The age of the patient at the time of FASCIA analysis, gender, diagnoses, and use of immunosuppressants were recorded. We categorized the patients in two groups: combined immunodeficiency (CID) or non-CID patients. The CID group consisted of patients with a diagnosed SCID, leaky SCID, or combined immunodeficiency. We also included in the CID group patients with syndromes where immunodeficiency is a known feature, such as cartilage hair hypoplasia and CHARGE syndrome if clinical symptoms of combined immunodeficiency (recurrent severe/abnormal infections, recurrent infections and malignancies, or stem cell transplantation) were present. Also, patients diagnosed with an immunodeficiency by the treating clinician and who had had opportunistic or recurrent deep infections were included in the CID group. Our classification of the patients was based on the updated classification of the International Union of Immunological Societies Expert Committee for Primary Immunodeficiency [1] and the European Society for Immunodeficiency’s clinical criteria [2]. Genetic diagnosis was available for 20 patients out of 30 in the CID group (see Supplementary Table S1A).

The non-CID cohort consisted of patients that were examined with lymphocyte stimulation test due to a clinical suspicion of an inborn error of immunity, but after all the immunological and other evaluations, and in some cases also follow-up time, primary immunodeficiency diagnosis was not made by the clinician. This heterogeneous patient group consisted of, for example, patients with less severe immunodeficiencies (such as specific antibody deficiency, antibody deficiencies), specific lung or skin presentations (e.g., severe atopy), premature infants, pediatric patients with an episode of protein losing enteropathy, and patients with a history of severe, recurrent, or abnormal infections. Forty percent of this group did not receive any specific primary immunodeficiency diagnosis after the examinations carried out by the responsible clinician. See Supplementary Table S1B for more detailed description of diagnoses included in the non-CID group.

### FASCIA Method Description

In the FASCIA method, diluted whole blood is stimulated for seven days with three mitogens ConA, phytohemagglutinin (PHA), and pokeweed mitogen (PWM) as described in detail before [8]. Minimal blood volume requirement for the test is 0.5 ml. We used the following mitogens and final concentrations in RPMI media (supplemented with 2 mM L-glutamine, HEPES, 100 IU/ml penicillin, and 100 IU/ml streptomycin): PHA 10 μg/ml, ConA 10 μg/ml, and PWM 5 μg/ml (all mitogens from Sigma-Aldrich, USA). PHA and ConA are primarily T cell mitogens, and PWM is widely used to stimulate B cells. Our laboratory has tested that samples can be stimulated after approximately 27 h storage or transport at room temperature without compromising the results (data not shown).

After the incubation, red cells were lysed with IOTest 3 Lysing Solution (Beckman Coulter, USA) and stained with following directly conjugated antibodies: CD8 PerCP-Cy5.5 (clone SK1), CD19 APC (clone SJ25C1), and BD Simultest CD3-FITC/CD4-PE (clones SK7 and SK3 respectively), all antibodies from BD Biosciences, USA. Flow cytometry data was acquired with FACS Canto equipment (BD Biosciences), and the data analyzed with FACS Diva software (BD Biosciences).

### FASCIA Method Validation

The FASCIA test was validated with standard laboratory validation before it was accepted into clinical use. Thymidine incorporation was in routine clinical use at our laboratory prior to the introduction of FASCIA in February 2015. The FASCIA test was validated with standard laboratory validation protocol before it was approved into clinical use prior to which it had already been validated against thymidine incorporation by Marits et al. [8]. Shortly, to compare these two assays, 44 patient samples and 28 healthy control samples were analyzed with both methods. In the evaluation, overall interpretation was used for comparison because due to the fundamental differences between these methods, quantitative results could not be compared. Based on approximately 70% positive concordance between the methods in the overall interpretation of the results as abnormal or normal, FASCIA was introduced to clinical diagnostic use (data not shown). In addition, the reproducibility of stimulation with FASCIA was tested with three patients and one control sample which were stimulated as duplicates, stained, and analyzed. The correlation coefficient between duplicate samples was 0.925.

During validation, we decided to analyze the percentage of activated lymphocytes after stimulation instead of absolute numbers of activated cells. Analysis of the percentages is technically less complicated and is a more robust measurement for patient samples since lymphopenia does not affect the results provided there is still a sufficient number of cells to be analyzed. We used 100 cells as a cut-off in the population of interest to be sufficient for analyzing the percentage of activated cells. The activated cells can be separated from the non-activated lymphocytes by flow cytometry based on their bigger size detectable with the forward scatter parameter available in all standard flow cytometers [9]. The main adaptive lymphocyte subtypes are separated by their surface antigens (CD3 + T cells and CD19 + B cells). Helper T cells are identified with surface staining for CD4. The surface marker for CD8 + T lymphocytes was added to the test in 2016. Prior to that, the lymphocytes were divided into CD4 + and CD4-, where the latter population mostly consists of CD8 + lymphocytes.

### FASCIA Data Analysis

All the FASCIA test results were re-analyzed by two clinical immunologists (A.T. and E.K.) for our study to ensure that the interpretation of the flow cytometer data was as comparable as possible. A representative gating strategy used for FASCIA analysis is shown in Supplementary Fig. 1a–d (a control sample unstimulated and stimulated with ConA, PHA, or PWM). Dead cells were excluded from the analysis based on their size and granularity. Propidium iodide (PI) staining of unstimulated, PHA-stimulated, and ConA-stimulated sample demonstrate that there is only a very small fraction of contaminating dead cells within lymphocyte gate (i.e., cells included in the analysis). These results suggest that dead cells do not significantly interfere with the interpretation of the results (Supplementary Fig. 2).

### Setting Reference Values for FASCIA

Stimulation results from 177 healthy adult blood donors were used for the FASCIA reference limit determination during laboratory clinical validation. Age and sex were available for 96 healthy controls. The median age of controls was 55 years (range 19–70 years). A total 64 of the controls were male (66.7%), and 32 were female (33.3%). The outliers were identified and removed from the data by Dixon’s criteria [10]. Lower reference limits (5.0 percentile of the distribution, 95% confidence intervals) were established for each mitogen stimulation using the non-parametric approach according to the Clinical and Laboratory Standards Institute’ guidelines. See Supplementary Table 2 for the reference limits used by our laboratory. The clinical immunologist interprets the results based on all stimulation responses, and if only one mitogen response is abnormal, that is not considered clinically significant.

### Statistical Analyses

Three-group comparisons were done with the Kruskal–Wallis test. Pairwise analyses were made using the Dunn–Bonferroni test. Receiver operator curve (ROC) analysis was used to evaluate the diagnostic performance and to assess the best cut-off value for the results. Null hypothesis was set at AUC 0.5 for calculating the *p* value for ROC. Statistical significance was set at *p* = 0.05. Statistical analyses were done with the IBM SPSS Statistics program (USA). Calculations for reference values were done using MS Excel and GraphPad Prism (USA).

## Results

### Patient Characteristics

Demographic details and basic immunological parameters of the two study populations are shown in Table 1. We included the lymphocyte counts measured within a month of the FASCIA testing to the analysis. The CID group had a lower mean lymphocyte count and lower total CD3-positive T cells. This is a logical finding considering that T cell lymphopenia is one of the diagnostic criteria for CID. The age distribution of the two patient groups was similar (Supplementary Fig. 3).

**Table 1:** Patient characteristics in the CID and non-CID groups

	CID (*n* = 30)	Non-CID (*n* = 159)	*p* value
Median age (years)	7	10.5	*p* = 0.57
Females/males (% of males)	16/14 (46.7%)	66/93 (58.5%)	*p* = 0.23
Mean leukocyte count (E9/l. ± SD)	6.8 ± 3.1	7.7 ± 4.3	*p* = 0.54
Mean lymphocyte count (E9/l ± SD)	2.1 ± 2.0	3 ± 2.6	*p* = 0.02
Mean CD3 + lymphocyte (T cell) count (E9/l ± SD)	1.3 ± 1.6	2.1 ± 1.8	*p* = 0.001
Mean CD19 + lymphocyte (B cell) count (E9/l ± SD)	0.6 ± 0.8	0.7 ± 0.9	*p* = 0.177
Immunosuppressive medication *n* (%)	5 (16.7%)	19 (11.9%)	*p* = 0.55

### Patients Diagnosed with Combined Immunodeficiency Have a Significantly Lower FASCIA Score

The FASCIA result in our laboratory is given as the percentages of lymphocytes responding to mitogen stimulation. As the final result, a percentage of stimulated cells is given separately for CD4-positive and CD8-positive T cells for all the three mitogens (prior adding the CD8 antibody to the panel, the results were given for the CD4-positive and CD4-negative cells), and PWM responses include an additional analysis of the CD19-positive B cells. Supplementary Table 3A summarizes the mean percentages of responsive lymphocytes of the total lymphocyte population. When looking at the individual mitogens, the variation in the results was quite large. There was especially variation in the CID group’s stimulation responses but also in the non-CID patient group. In the healthy controls, the widest range in the stimulation responses was seen after PWM stimulation. We redid the analysis with CID and non-CID patients on immunosuppressive medications excluded, which reduced the spread in the stimulation responses (Supplemental Table 3B).

In order to simplify the analysis and interpretation, we formed a score from the FASCIA results by calculating the mean of percentages of activated cells to all mitogens used. Therefore, the values of FASCIA score ranged between 0 and 100.

**Fig. 2:**
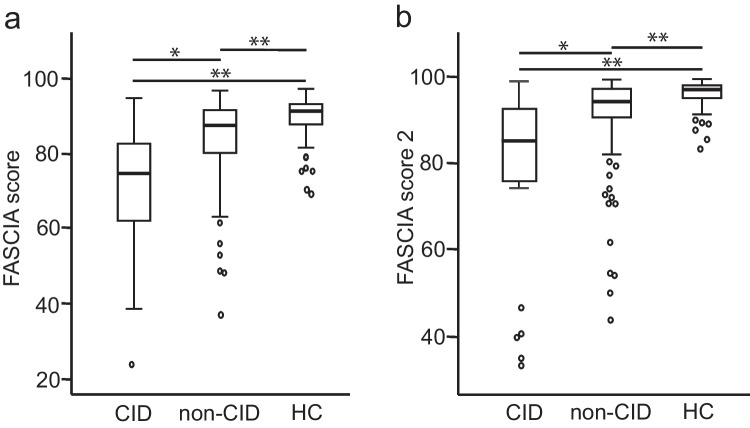
**a** The FASCIA score was significantly lower in the CID group compared to the non-CID group (70.5 vs 84.1, *p* = 0.002). Also the score was lower in the non-CID group than in the healthy controls (84.1 vs 89.6, *p* < 0.001). **b** FASCIA score 2 without the PWM stimulation differed also significantly between the CID and non-CID patients (78.0 vs 91.5, *p* = 0.001) as well as between the non-CID patients and the controls (91.5 vs 96.2, *p* < 0.001). * *p* < 0.003, ** *p* < 0.001

The FASCIA score was significantly lower in the CID group compared to the non-CID group (70.5 vs 84.1, *p* = 0.002) (Fig. 2a). Also, in the non-CID group, the score was lower than the healthy controls (84.1 vs 89.6, *p* < 0.001) (Fig. 2a). We then formed another score (FASCIA score 2) that did not include the PWM mitogen which showed the biggest variation even among the healthy controls. Also this score differed significantly between the CID and non-CID patients (78.0 vs 91.5, *p* = 0.001) as well as between the non-CID patients and the controls (91.5 vs 96.2, *p* < 0.001) (Fig. 2b).

Since the lower FASCIA score could be a result of T cell lymphopenia in the CID cohort, we tested if there was a correlation between the score and absolute T cell count. The T cell number and the FASCIA score did not have a correlation with each other (correlation coefficient − 0.043 with Spearman`s rank correlation test) (Supplementary Fig. 4). This result of no correlation between T cell counts and the FASCIA score further supports the use of percentages of activated cells instead of absolute numbers as the readout of the FASCIA method.

### FASCIA Shows Good Diagnostic Performance in Identifying CID

**Fig. 3:**
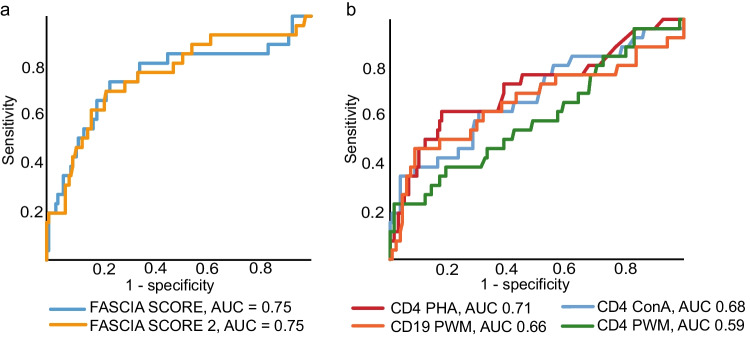
**a** In the ROC analysis, the AUC was 0.75 (*p* < 0.001) for both the FASCIA score (blue) and FASCIA score 2 (yellow). **b** Of the individual mitogens PHA (red) performed best in separating patients with CID from the rest (AUC 0.71, *p* < 0.001). ConA stimulation for CD4 positive T cells had an AUC of 0.68 (blue, *p* = 0.004). For CD4 + T cell stimulation with PWM (green), the AUC was only 0.59 (*p* = 0.14). Also for the CD19 + B cells, the PWM stimulation had a low AUC of 0.66 (orange, *p* = 0.011)

ROC analysis is used to evaluate diagnostic performance of a diagnostic test [11]. In the ROC analysis, the area under the curve (AUC) was 0.75 (*p* < 0.001) for both the FASCIA score and FASCIA score 2 (Fig. 3a). Generally, AUC values 0.7–0.8 are considered acceptable for a diagnostic screening test [11].

Of the three mitogens studied separately, PHA stimulation response of CD4-positive T cells performed best in identifying patients with CID from the whole tested patient cohort (AUC 0.71, *p* < 0.001) (Fig. 3b). The cut-off of 93% (near our laboratory’s reference values lower limit) of activated CD4-positive cells in PHA stimulation had a sensitivity of 0.6 and specificity of 0.2 for CID diagnosis.

PWM response showed a wide variation even among healthy controls (Supplementary Table 3A), and the proportion of activated population for this mitogen in the CD4-positive cell population did not separate between the CID and non-CID patients (AUC 0.59, *p* = 0.14) (Fig. 3b). For the CD19-positive B cells also, the PWM stimulation in FASCIA test performed only modestly in identifying the CID patients from the whole cohort tested (AUC 0.66, *p* = 0.011).

We estimated the sensitivity and specificity of our laboratory established reference values from the ROC analysis. A FASCIA score of the lower reference limits was 72. Using that as a cut-off, the sensitivity of the test to correctly identify CID was 0.4 and 1 specificity 0.099. FASCIA score 2 for lower reference limits of ConA and PHA stimulations is 83, and its sensitivity was 0.46 and 1 specificity 0.1.

### Immunosuppressive Medication Has a Negative Effect on the FASCIA Score

Of the 189 study subjects, 24 were on immunosuppressive medication at the time of FASCIA analysis. Of these, 19 were in the non-CID and 5 in the CID group. Additionally, 8 subjects used topical tacrolimus and/or large amounts of high-grade topical steroids that were thought to render a systemic impact (all in the non-CID group) and 21 used inhalation steroids (17 in the non-CID group). In some of the latter, the steroid dose was large enough for a systemic effect. Because the systemic effect of topical or inhaled agents cannot be verified, we only analyzed the influence of systemic immunosuppressive medication on the FASCIA score. Individual patients used azathioprine, cyclosporine, mycophenolate, and biological drugs (adalimumab, infliximab, or tocilizumab).

**Fig. 4:**
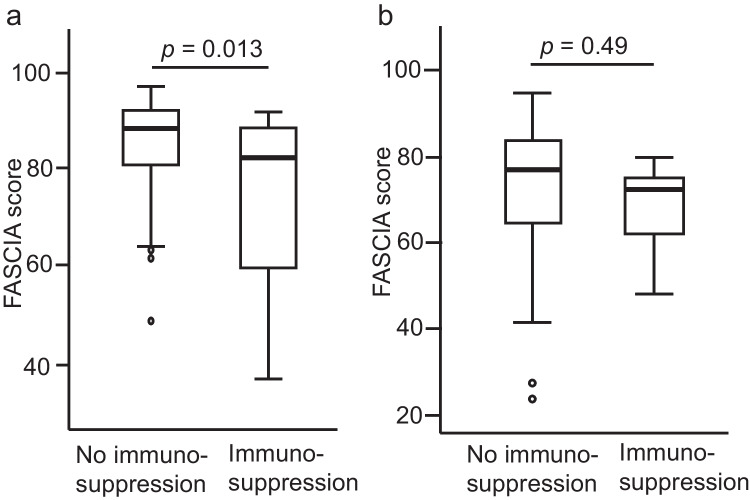
**a** The FASCIA score among the non-CID patients with immunosuppressive medication (*n* = 15) was lower than among the non-medicated (*n* = 127) (*p* = 0.013). **b** The FASCIA score was also lower in the medicated CID patients compared to the non-medicated CID patients, but the difference was not statistically significant (*p* = 0.49)

The FASCIA score among the medicated non-CID patients (*n* = 15) was lower than among the non-medicated (*n* = 127) (*p* = 0.013) (Fig. 4a). Five subjects in the CID group also had immunosuppressive therapy: one used prednisolone, one adalimumab, two tacrolimus combined with prednisolone, and one prednisolone combined with hydroxychloroquine. The FASCIA score was lower in the medicated group compared to the non-medicated CID patients, but the difference was not statistically significant (*p* = 0.49) (Fig. 4b).

## Discussion

Even though affordable high-throughput sequencing has revolutionized the diagnostics and our understanding of the inborn errors of immunity within the past decade, functional testing still has its role in the primary diagnostics of these diseases. It is also essential in evaluating the potential pathology of new variants of unknown significance. In measuring lymphocyte function, FASCIA has many benefits compared to thymidine incorporation or CFSE which have been used in the diagnostics for CID. One of the most important of these from the perspective of a clinical immunology laboratory is avoiding the use of radioactive reagents.

Here, we show that FASCIA can be used in the diagnostics of CID together with other laboratory parameters, clinical criteria, and genetic analysis. Our finding is in line with the previous work done with SCID patients [8]. However, the method is not reliable when systemic immunosuppressive medication is used, and this should be kept in mind when interpreting the test result.

In our study, the FASCIA results varied also among the CID patients. Even though a severely diminished response of the lymphocytes to mitogens is one the diagnostic criteria of SCID, it has already previously been shown that depending on the genetic background of the disease, the response to PHA varies and may be close to normal [12]. This highlights the complexity of the diagnostics of inborn errors of immunity with no single test detecting these diseases accurately.

Of the individual mitogens, PHA was the best in separating patients with CID from the non-CID patients. In a previous study where thymidine incorporation was used to measure lymphocyte proliferation, ConA was superior to PHA [4]. Yet, in most instances, PHA is used as the principal mitogen in measuring lymphocyte function, and our data suggest that this is justified in clinical practice. PWM had the largest variation even among the healthy controls in our study. When looking only at the T cell proliferation, PWM could be left out of the FASCIA test, making the test even simpler and more cost efficient. Overall, the FASCIA score performed slightly better than the best individual mitogen PHA which supports that the clinical interpretation of the lymphocyte stimulations should preferably be done based on several abnormal stimulation responses. Our laboratory’s reference values favored specificity over sensitivity for CID diagnosis. For a test like FASCIA, it is impossible to give exact cut-off values since the population and diagnoses studied are so variable. Moreover, for a functional lymphocyte test, it is vital that the laboratory performing the test establishes their own reference values and maintain good technical controls since no international standardization exists [13].

Most patients in our cohort were pediatric, but the age variation was large. However, the age distribution did not significantly differ between the two study groups. A clear limitation of our study is the lack of pediatric reference values. This potentially underestimates the immunodeficiency in many pediatric patients [4]. It has been proven already decades ago that lymphocyte proliferation in response to mitogens declines with increasing age [14]. Therefore, if we had had reference values from age-matched healthy controls, the difference between the CID and non-CID patients could have been even more clear-cut. Acquiring blood samples from healthy children poses an ethical challenge, and we did not have the opportunity to include healthy pediatric samples into our analysis. Another major limitation of his study was its retrospective nature and the following heterogeneity of the study population. On the other hand, our cohort reflects the heterogeneity of the real-life patients evaluated for suspected inborn errors of immunity and highlight how the FASCIA method performs in real-life clinical laboratory practice in identifying CID patients.

Our study population was sizable compared to previously published data. It has been shown that FASCIA works in SCID diagnostics, but the previous study included only a few individual patients [8]. We had a fair amount of other CID patients besides SCID showing that the test can be used also in a wider spectrum of immunodeficiency diseases. The non-CID population included both milder immunodeficiencies as well as patients with other diseases and normal immunology, accurately reflecting the real-life population from which physicians need to identify the CID patients. CID diagnosis is based on several criteria, of which T cell lymphopenia and reduced T cell activation to mitogen stimulus are central. We could show here that the FASCIA score did not correlate with the T cell lymphopenia emphasizing its value as an independent laboratory metric in the CID diagnostic algorithm.

## Conclusions

In conclusion, FASCIA can reliably detect the CID patients from a population tested for suspected immunodeficiency if no immunosuppressive medication is used. The main benefit of this method compared to thymidine incorporation is that there is no need for radioactive reagents. FASCIA requires small sample volume making it ideal when examining pediatric patients. Our results show that FASCIA is a valuable option as a practical laboratory method for measuring lymphocyte stimulation responses. It is easy to perform and analyze. We conclude that FASCIA is a versatile method suitable for not only immunodeficiency diagnostics but also for other functional in vitro testing of T lymphocytes [15].

## Supplementary Information

Below is the link to the electronic supplementary material.

Supplementary file1 (PDF 410 KB)

## Data Availability

The datasets generated and analyzed during the current study are available from the corresponding author on reasonable request. The dataset consists of information on patients and thus cannot be publicly available due to the General Data Protection Regulation.
