# Supplementary material for: FASCIA Method in the Assessment of Lymphocyte Mitogen Responses in the Laboratory Diagnostics of Primary Immunodeficiencies
Source: J Clin Immunol. 2022 Dec 13;43(3):653–61. doi: 10.1007/s10875-022-01417-z (PMC9958160; doi:10.1007/s10875-022-01417-z)
Supplement: Supplementary file 1 — Supplementary file1 (PDF 410 KB) [file 10875_2022_1417_MOESM1_ESM.pdf]

Supplementary table 1A: Detailed diagnoses included in the CID group.

Gene affected / syndrome / clinical basis for CID diagnosis	No of patients
RTEL1	1
Cartilage-hair hypoplasia	5
TTC7A	1
STAT1 (GoF)	3
STAT3 (GoF)	1
TOM1	1
JAK3 (compound heterozygous)	1
IL2Rg (X-linked)	1
RFX5 (compound heterozygous)	1
CHD7 (CHARGE)	1
MHC class II deficiency (unidentified gene defect)	1
DNA ligase IV deficiency	1
RAG1	1
RAG2	1
STAT3 (LoF)	1
Clinical diagnosis of CID (opportunistic infections, severe bacterial and viral infection, colitis, malignancy)	9
Total	30

Supplementary table 1B: Groups of diagnoses included in the non-CID group.

	No of patients
Syndromes (with immunodeficiency component not fulfilling CID diagnostic criteria or with no immunodeficiency)	39
Antibody deficiencies	21
Other immune deficiency diagnosis (eg. complement deficiency)	5
Patients with autoimmune disease	8
Patients that had received a stem cell transplantation	11
Patients with no immunological diagnosis during the study period	75
Total	159

Supplementary Table 2. Reference limits based on 177 healthy adult blood donors.

Stimulation	Lower ref. limit (95 CI)
CD4 PHA	92% (88-94)
CD8 PHA	87% (75-92)
CD4 ConA	77% (70-81)
CD8 ConA	76% (58-79)
CD4 PWM	67% (41-73)
CD8 PWM	54% (39-59)
CD19 PWM	48% (30-53)

Supplementary table 3A: Mean percentages of responsive lymphocytes of total lymphocytes for CD19+ and of CD3+ cells for T cell subpopulations. Antibody for CD8 + cells was added to the analysis 2016, therefore prior to that the T-cell population was divided to CD4+ and CD4- cells, the latter consisting mostly of CD8+ cells.

Mean % of responsive cells to a mitogen	CID	non-CID	HC	*p* value CID vs HC	*p* value non-CID vs HC	*p* value CID vs non-CID
CD4+ ConA (±SD; range)	69.6 (33.3; 0.0-99.3)	87.7 (13.6; 10.1-99.5)	93.5 (4.7; 69.8-99.3)	0.002	0.027	0.222
CD8+ ConA (±SD; range)	64.1 (35.6; 1.0-99.3)	85.0 (18.7; 2.6-99.6)	93.6 (4.9; 76.9-99.1)	0.008	<0.001	0.196
CD4- ConA (±SD; range)	74.2 (26.5; 12.1-98.9)	90.3 (12.4; 25.7-99.2)	95.2 (3.0; 86.2-99.0)	<0.001	0.162	0.007
CD4+ PHA (±SD; range)	85.6 (16.3; 36.4-99.4)	94.8 (7.6; 52.5-99.9)	99.0 (1.0; 91.9-99.9)	<0.001	<0.001	0.050
CD8+ PHA (±SD; range)	90.5 (12.5; 57.1-99.4)	95.2 (6.8; 48.8-99.8)	98.4 (2.0; 81.3-99.8)	0.507	<0.001	1.000
CD4- PHA (±SD; range)	87.0 (14.0; 46.4-99.4)	96.3 (4.1; 78.2-99.4)	98.7 (0.7; 96.5-99.7)	<0.001	<0.001	0.005
CD4+ PWM (±SD; range)	65.0 (29.6; 1.4-97.5)	78.0 (15.3 ; 13.1-99.0)	84.8 (9.4; 20.2-96.1)	0.068	<0.001	0.981
CD8+ PWM (±SD; range)	52.2 (29.1; 1.0-84.6)	73.5 (19.2; 9.1-94.5)	75.7 (11.5; 36.0-93.4)	0.223	1.0	0.048
CD4- PWM (±SD; range)	56.0 (25.9; 16.9-94.2)	67.7 (16.7; 24.4-96.4)	69.4 (11.0; 45.585.0)	0.001	<0.001	0.379
CD19+ PWM (±SD; range)	56.2 (29.1; 10.8-100.0)	73.2 (20.8; 5.9-98.8)	84.2(13.9; 24.6-99.2)	0.006	<0.001	0.065
FASCIA score (±SD; range)	70.5 (18.3; 23.5-94.3)	84.1 (10.4; 36.5-96.3)	89.6 (4.7; 68.6-96.8)	<0.001	<0.001	0.002
FASCIAscore2 (±SD; range)	78,0 (20,8; 32,9-98,8)	91,5 (9,6;43,3-99,2)	96,2(2,5; 83,2-99,3)	<0.001	<0.001	0.001

CID = combined immunodeficiency, non-CID = no CID diagnosis, HC = healthy control

Supplemetary Table 3B: Mean percentages of responsive lymphocytes of the total lymphocytes. In the CID and non-CID group patients with immunosuppressive medication were excluded.

Mean % of responsive cells to a mitogen	CID	non-CID	HC	*p* value CID vs HC	*p* value non-CID vs HC	*p* value CID vs. non-CID
CD4+ ConA (±SD; range)	70.3 (33.2; 0-97.4)	89.1 (10.2; 50.1-99.5)	93.5 (4.7; 69.8-99.3)	0.009	<0.001	0.008
CD8+ ConA (±SD; range)	64.1 (35.6; 1.0-99.3)	88.2 (12.6; 32.4-99.6)	93.6 (4.9; 76.9-99.1)	<0.001	0.001	0.042
CD4- ConA (±SD; range)	77.7 (23.7; 20.0-98.9)	90.0 (12.6; 25.7-99.2)	95.2 (3.0; 86.2-99.0)	0.004	0.130	0.110
CD4+ PHA (±SD; range)	84.5 (17.5; 36.4-99.4)	95.5 (6.4; 61.4.99.9)	99.0 (1.0; 91.9-99.9)	<0.001	<0.001	0.061
CD8+ PHA (±SD; range)	90.5 (12.5; 57.1-99.4)	96.3 (4.8; 75.2-99.8)	98.4 (2.0; 81.3-99.8)	0.011	<0.001	0.908
CD4- PHA (±SD; range)	87.3 (16.0; 46.4-99.4)	96.3 (4.2; 78.2-99.4)	98.7 (0.7; 96.5-99.7)	<0.001	<0.001	0.099
CD4+ PWM (±SD; range)	65.9 (31.6; 1.4-97.5)	78.7 (14.5; 13.1-99.0)	84.8 (9.4; 20.2-96.1)	0.012	0.001	0.97
CD8+ PWM (±SD; range)	52.2 (17.4; 15.4-94.5)	75.3 (17.4; 15.4-94.5)	75.7 (11.5; 36.0-93.4)	0.012	0.785	0.003
CD4- PWM (±SD; range)	56.8 (26.3; 16.8-94.2)	68.5 (16.3; 24.4-96.4)	69.4 (11.0; 45.585.0)	>0.05	>0.05	>0.05
CD19+ PWM (±SD; range)	59.1 (30.4; 10.8-100.0)	74.6 (19.3; 17.2-98.8)	84.2 (13.9; 24.6-99.2)	<0.001	<0.001	0.475
FASCIA score (±SD; range)	70.8 (20.7; 23.5-94.3)	85.2 (8.5; 48.4-96.3)	89.6 (4.7; 68.6-96.8)	<0.001	<0.001	0.016
FASCIA score2 (±SD; range)	78.1 (21.0; 32.9-98.8)	92.6(7.2; 53.9-99.2)	96.2(2.5; 83.2-99.3)	<0.001	<0.001	0.001

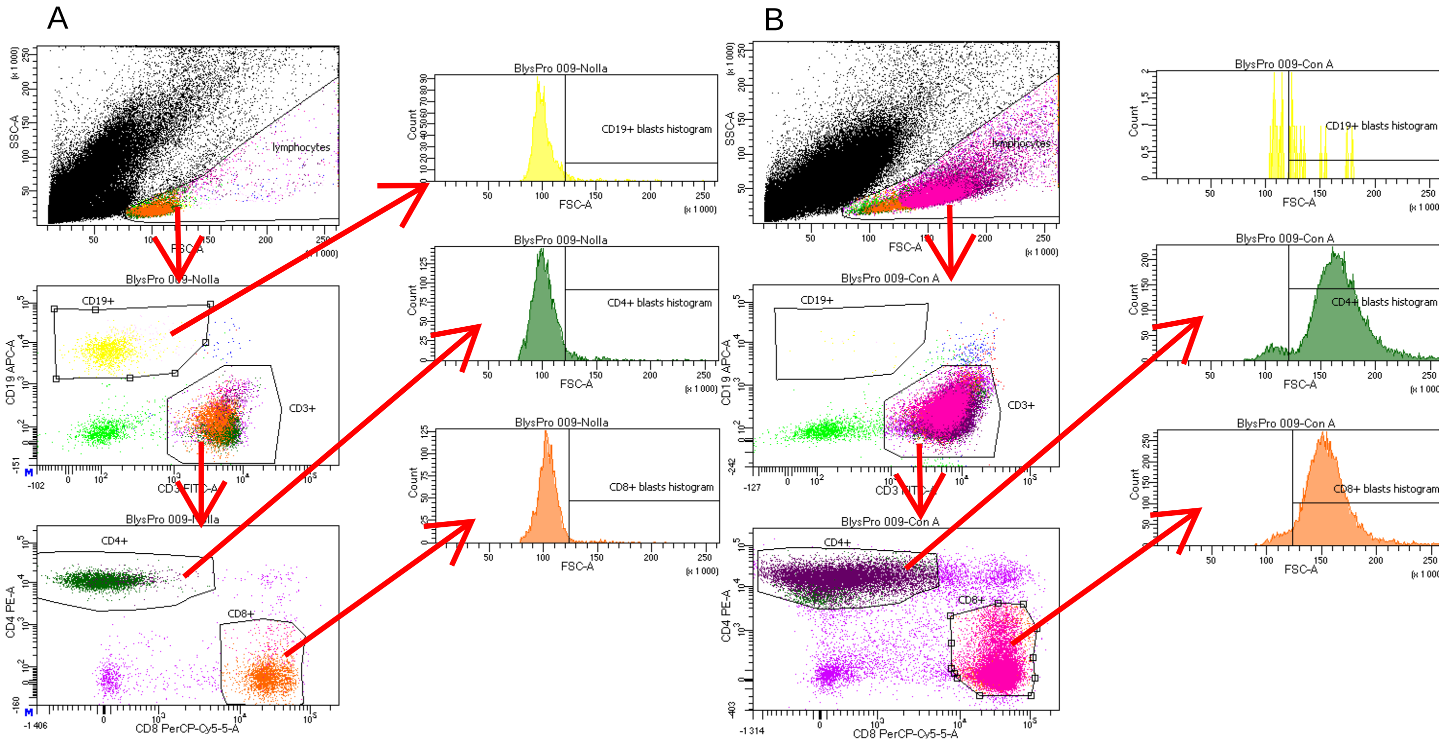

Supplementary Figure 1: A) Representative gating strategy for a unstimulated control sample. B) representative gating strategy for a ConA-stimulated control sample. C) Representative gating strategy for a PHA-stimulated control sample. D) Representative gating strategy for a PWM-stimulated control sample. CD19-positive B cell stimulation response is only analysed in the PWM-stimulation.

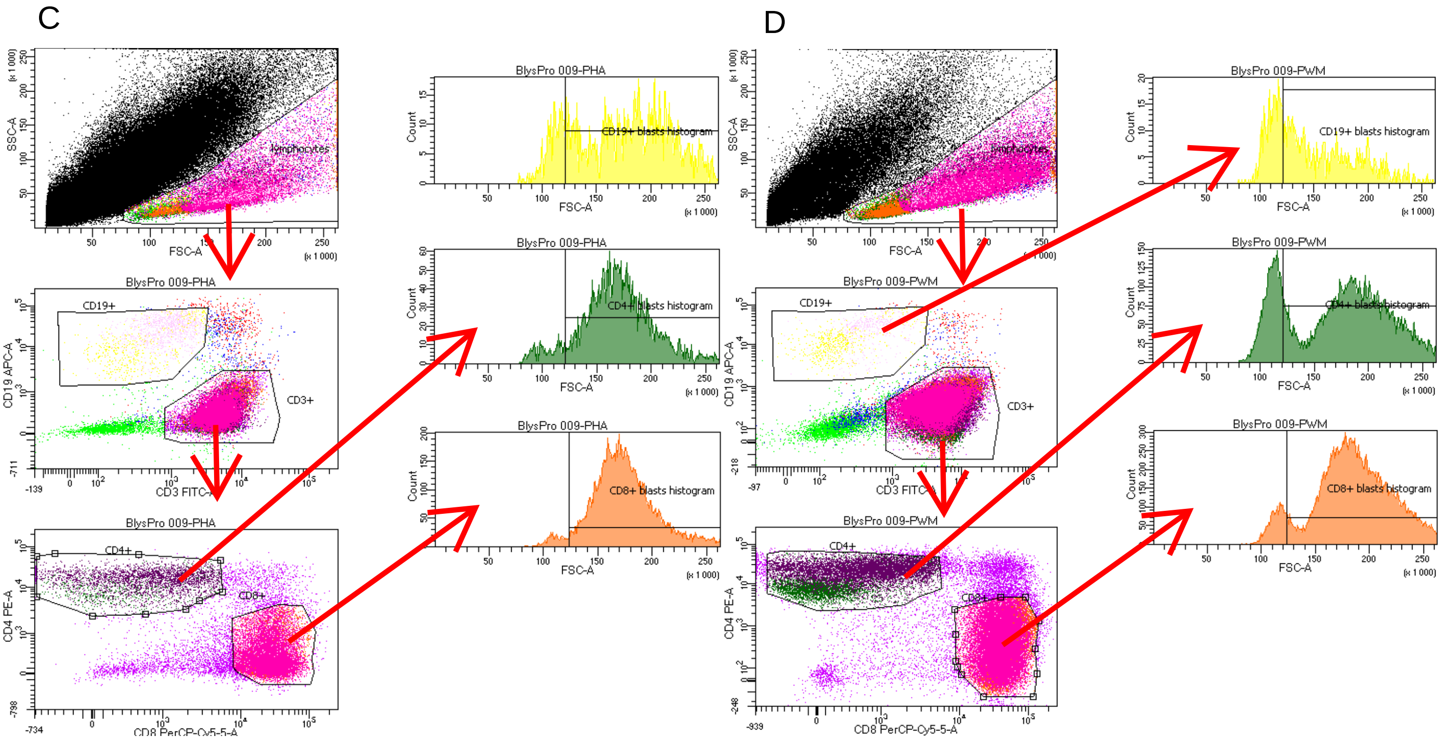

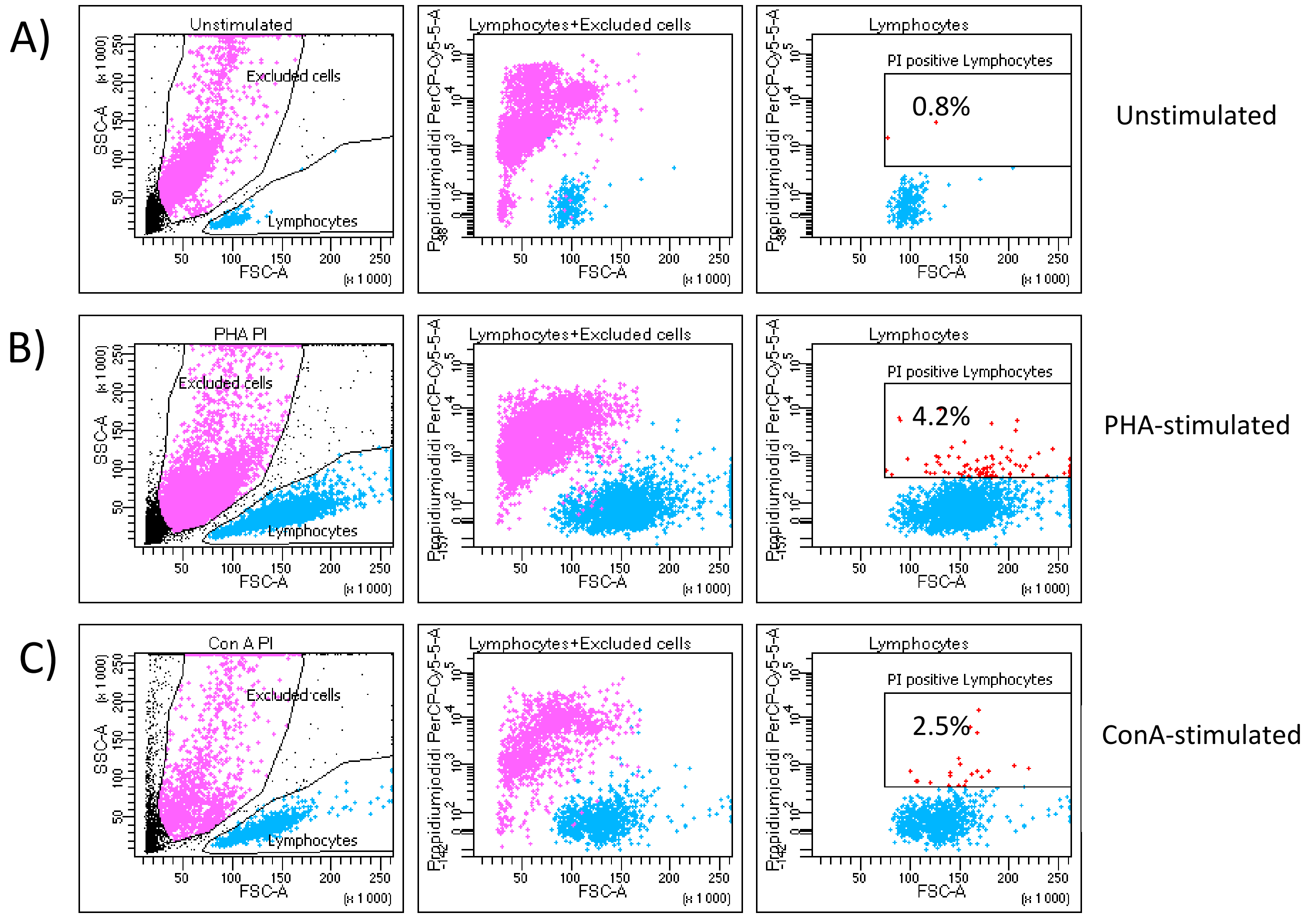

Supplementary Figure 2.

Representative dot plots of Propidium iodide-staining of A) Unstimulated B) PHA-stimulated and C) ConA-stimulated sample. Majority of the cells within Lymphocytes –gate stain negative for propidium iodide and can be considered as living cells.

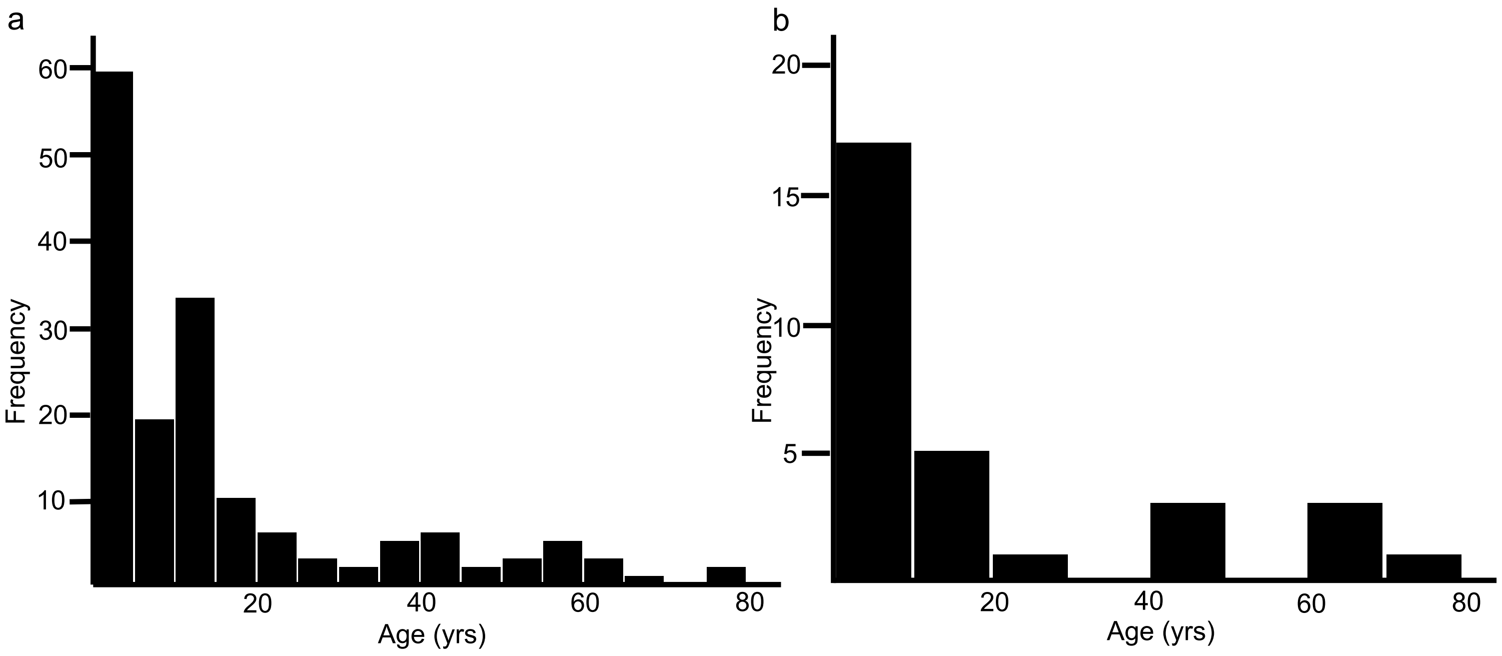

Supplementary Fig 3: Histogram of the patients' ages in a) non-CID and b) CID groups.

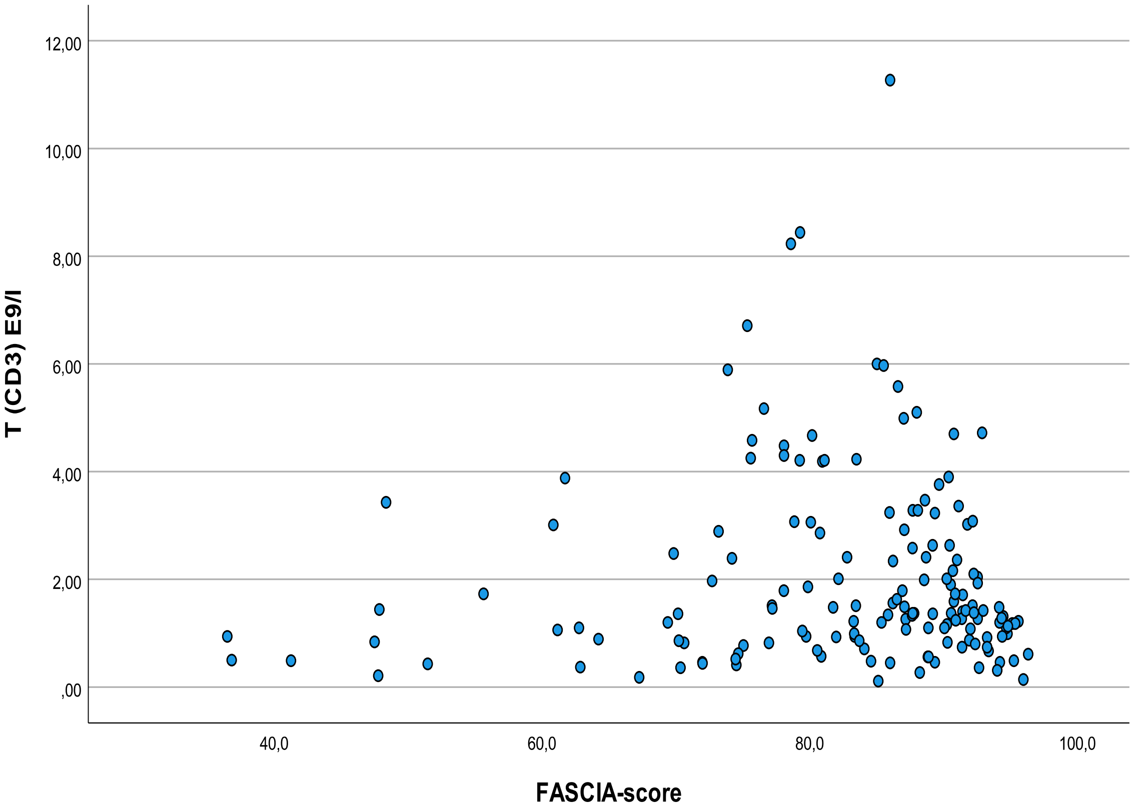

Supplementary Figure 4: Scatter plot of absolute T cell counts and the FASCIA score for patients that had both values available (n=159). Correlation coefficient -0.043 with Spearman`s rank correlation test.
